# PeCHYR1, a ubiquitin E3 ligase from *Populus euphratica*, enhances drought tolerance via ABA‐induced stomatal closure by ROS production in *Populus*

**DOI:** 10.1111/pbi.12893

**Published:** 2018-03-08

**Authors:** Fang He, Hou‐Ling Wang, Hui‐Guang Li, Yanyan Su, Shuang Li, Yanli Yang, Cong‐Hua Feng, Weilun Yin, Xinli Xia

**Affiliations:** ^1^ Beijing Advanced Innovation Center for Tree Breeding by Molecular Design National Engineering Laboratory for Tree Breeding College of Biological Sciences and Technology Beijing Forestry University Beijing China

**Keywords:** drought tolerance, stomatal closure, abscisic acid, *PeCHYR1*, *Populus*

## Abstract

Drought, a primary abiotic stress, seriously affects plant growth and productivity. Stomata play a vital role in regulating gas exchange and drought adaptation. However, limited knowledge exists of the molecular mechanisms underlying stomatal movement in trees. Here, *PeCHYR1*, a ubiquitin E3 ligase, was isolated from *Populus euphratica*, a model of stress adaptation in forest trees. *PeCHYR1* was preferentially expressed in young leaves and was significantly induced by ABA (abscisic acid) and dehydration treatments. To study the potential biological functions of *PeCHYR1*, transgenic poplar 84K (*Populus alba* × *Populus glandulosa*) plants overexpressing *PeCHYR1* were generated. *PeCHYR1* overexpression significantly enhanced H_2_O_2_ production and reduced stomatal aperture. Transgenic lines exhibited increased sensitivity to exogenous ABA and greater drought tolerance than that of WT (wild‐type) controls. Moreover, up‐regulation of *PeCHYR1* promoted stomatal closure and decreased transpiration, resulting in strongly elevated WUE (water use efficiency). When exposed to drought stress, transgenic poplar maintained higher photosynthetic activity and biomass accumulation. Taken together, these results suggest that *PeCHYR1* plays a crucial role in enhancing drought tolerance via ABA‐induced stomatal closure caused by hydrogen peroxide (H_2_O_2_) production in transgenic poplar plants.

## Introduction

Drought stress is a common abiotic stress, affecting plant growth, seed production, gas exchange, water relations and cellular homeostasis in plants (Shinozaki and Yamaguchi‐Shinozaki, 2007; Zhu, 2016). To adapt to hostile environments, plants have developed intrinsic mechanisms to mitigate drought stress, such as closing stomata, reducing transpiration, generating abscisic acid (ABA) and accumulating hydrogen peroxide (H_2_O_2_) (Dietz *et al*., 2016; Zhu, 2016). ABA‐induced stomatal closure is a defensive action that is usually accompanied by the production of H_2_O_2_ (Li *et al*., 2017; Zhang *et al*., 2001). NADPH oxidase (RbohF) can generate H_2_O_2_ in guard cells, which crucially modulates ABA‐induced stomatal closure and plant drought tolerance (Gudesblat *et al*., 2007; Kolla *et al*., 2007; Ma *et al*., 2016). Previous studies have indicated that AtRbohF can be phosphorylated by OST1 (an ABA‐induced SnRK2.6 protein kinase) (Joshi‐Saha *et al*., 2011; Sirichandra *et al*., 2009; Wege *et al*., 2014). H_2_O_2_ is a reactive oxygen species (ROS) that acts as a major signalling molecule in the ABA response pathway in guard cells (Bright *et al*., 2006; Li *et al*., 2017). High concentrations of ROS lead to cell injury or even hypersensitive cell death, whereas low concentrations of ROS function as developmental signals, controlling all aspects of plant biology (Ahmad *et al*., 2008; Dietz *et al*., 2016; Huang *et al*., 2016; Karuppanapandian *et al*., 2011; Pitzschke *et al*., 2006). OsASR5 and DCA1, which enhance drought tolerance in rice, control stomatal closure by adjusting the concentration of H_2_O_2_ (Cui *et al*., 2015; Li *et al*., 2017)_._ GHR1 modulates stomatal movement associated with H_2_O_2_ signalling in Arabidopsis (Hua *et al*., 2012).

In stomata, ABA‐mediated ROS generation elevates cytosolic calcium ion levels and gives rise to stomatal closure (McAinsh *et al*., 1996; Pei *et al*., 2000; Xing *et al*., 2008). Recently, it was reported that ROS can induce stomatal closure through an ABA‐independent pathway in rice (*Oryza sativa*). The SNAC1‐targeted gene OsSRO1c positively controls H_2_O_2_‐induced stomatal closure by regulating hydrogen peroxide level in rice, and the E3 ligase OsHTAS can promote heat tolerance by modulating H_2_O_2_‐induced stomatal closure in rice (Liu *et al*., 2016; You *et al*., 2013). However, 35S:*OsASR5* plants were more sensitive to exogenous ABA treatment, activating the production of H_2_O_2_ (Li *et al*., 2017).

RING (really interesting new gene) finger proteins consist mainly of conserved ‘CxHY’ domains (McDowall, 2007). These conserved ‘CxHY’ motifs are part of the CHY zinc‐finger domain, which plays a role in physical interaction and ubiquitination (Lee and Kim, 2011; Wooff *et al*., 2004; Yu *et al*., 2016), contains 12 histidines and cysteines, and binds with three zinc ions, composing a unique zinc‐finger motif (Cayrol *et al*., 2007; Ding *et al*., 2015; Esposito *et al*., 2006). Proteins such as ubiquitin (ubi) E3 ligases, a core component of ubiquitination pathway containing a CHY zinc‐finger domain, possess a universal function including ubiquitylation of substrate proteins (Lim *et al*., 2013a; Ning *et al*., 2016; Stone *et al*., 2005). The ubiquitination process plays a crucial role in plant development and response to environmental stresses (Lim *et al*., 2013a; Stone *et al*., 2005). RING ubi E3 ligases play decisive roles in almost all plant growth processes, including photomorphogenesis, flower development, phytohormone signalling and regulation of senescence (Bae *et al*., 2011; Ding *et al*., 2015; Lim *et al*., 2013b, 2017). RING E3 ligases have been found in diverse plants, such as hot pepper (*Capsicum annuum*), maize (*Zea mays*), Arabidopsis and rice, positively or negatively regulating abiotic stress (Lyzenga and Stone, 2012; Park *et al*., 2015; Zhao *et al*., 2014). Recently, a ubiquitin E3 ligase, Arabidopsis CHYR1, was found to positively facilitate ABA and drought‐mediated stomatal closure, ROS production and plant drought tolerance via SnRK2.6‐mediated phosphorylation (Ding *et al*., 2015). However, the expression of a homolog of *CHYR1*, rice *OsRZFP34*, enhances stomatal opening, leaf cooling and ABA insensitivity (Hsu *et al*., 2014).

Although the roles of CHYR1 family proteins have been widely reported in herbaceous plants, their possible functions in ligneous plants, especially in stomatal movement, remain unknown. Several mechanisms of plant response to drought exist, including avoidance, escape and tolerance strategies. Tolerance mechanisms attempt to maintain plant functions at the same level as in unstressed conditions, while avoidance mechanisms are used to adjust the balance between water loss and water uptake (Moradi, 2016). *Populus*, a pioneer genus in forest ecosystems, possesses great economic and ecological value (Sterky *et al*., 2004). Most poplar plants are fast‐growing and have poor drought tolerance (Larcheveque *et al*., 2011; Tuskan *et al*., 2006; Windt *et al*., 2006). However, one exception, *P. euphratica,* is the only large tree species that forms forests in desert areas, playing a very important role in the maintenance of the local ecological balance. Due to its strong adaptability to extreme temperature, drought and salinity, *P. euphratica* has served as a model woody plant to explore the mechanisms of stress resistance (Chen *et al*., 2013; Li *et al*., 2011; Ma *et al*., 2013; Tang *et al*., 2013; Yan *et al*., 2012). From an analysis of a transcriptome from *P. euphratica* under drought stres*s*, we selected *PeCHYR1*, which is up‐regulated in drought‐stressed *P. euphratica* leaves, and performed further functional analysis of this gene in poplar (Tang *et al*., 2013). In the current study, *PeCHYR1* was cloned from *P. euphratica* and transferred into poplar 84K (*P. alba* × *P. glandulosa*) (Feng *et al*., 2015; Ke *et al*., 2016). Next, we verified the function of *PeCHYR1* by means of molecular biology and plant physiological indexes. We found that *PeCHYR1* was a functional homolog of *CHYR1*. Overexpression of *PeCHYR1* enhanced drought tolerance by promoting H_2_O_2_‐mediated stomatal closure in poplar.

## Results

### Isolation and sequence analysis of *PeCHYR1*

**Figure 1:**
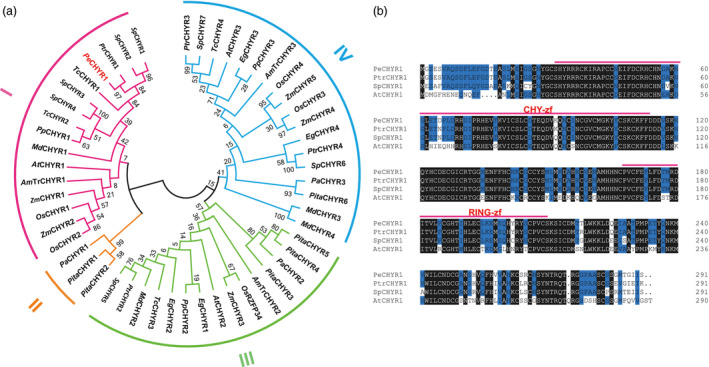
Amino acid sequence alignment and phylogenetic tree of different CHYR1 protein family members. (a) Phylogenetic analysis of the *PeCHYR1* homologs from *Populus*,* Amborella*,* Eucalyptus*,* Malus*,* Prunus*,* Theobroma*,* Picea*,* Pinus*,* Salix*,* Arabidopsis*, rice and maize. (b) Multiple alignment of the amino acid sequences of CHYR1 proteins from *Populus*,* Salix* and *Arabidopsis*.

To elucidate the potential function of *PeCHYR1* in the abiotic stress response of ligneous plants, we took advantage of *P. euphratica*, which naturally grows in deserts, to clone *PeCHYR1*. The coding sequence length of *PeCHYR1* was 876 bp, encoding 291 amino acids. Homology analyses of the amino acid sequence showed 52 homologous genes in *Populus*,* Amborella*,* Eucalyptus*,* Malus*,* Prunus*,* Theobroma*,* Picea*,* Pinus*,* Salix*,* Arabidopsis*, rice and maize. The evolutionary tree of related genes was divided into four parts (I, II, III and IV). The nucleic acid sequence of *PeCHYR1* is mostly identical to that of *PtrCHYR1* (Potri.009G005700), sharing 96.7% sequence identity (Figure 1a). A multiple sequence alignment revealed that the amino acid sequences of PeCHYR1, PtrCHYR1, SpCHYR1 and AtCHYR1 (AT5G22920) contained the same conserved domains (Figure 1b). Furthermore, the *PeCHYR1* promoter contains CAAT and TATA motifs that are involved in drought stress response (Figure S1).

### *PeCHYR1* involvement in ABA and water stress response

**Figure 2:**
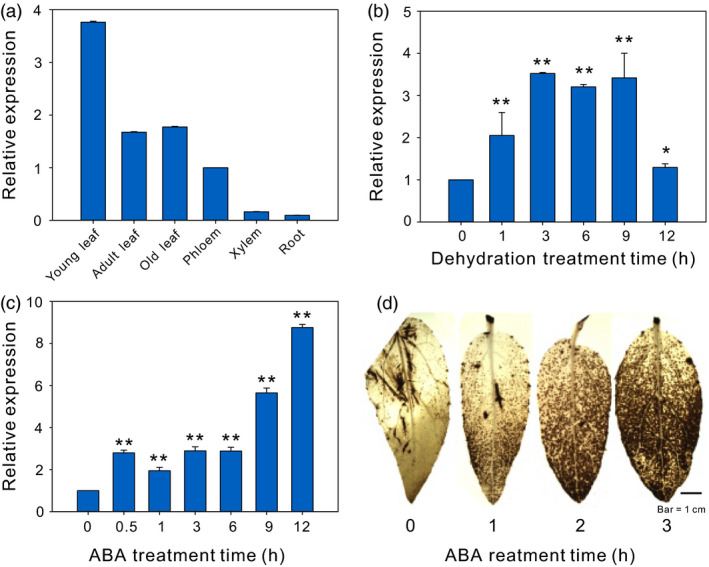
*PeCHYR1* expression patterns in different tissues and under different treatments. (a) Relative expression levels of the *PeCHYR1* gene in different tissues of *P. euphratica*. Young leaf; Adult leaf; Old leaf; Phloem; Xylem; Root. (b) Transcript levels of *PeCHYR1* were measured by RT‐qPCR in response to dehydration. (c) Transcript levels of *PeCHYR1* were measured by RT‐qPCR in response to ABA. (d) DAB staining in WT seedling leaves during ABA treatment. Error bars are means ± SE (*n *=* *20). Asterisks denote significant differences: *P <* *0.05; **P <* *0.01.

To study the potential biological functions of *PeCHYR1*, we applied quantitative real‐time polymerase chain reaction (RT‐qPCR) analysis to determine the relative transcript abundance in various tissues of *P. euphratica*. We observed that *PeCHYR1* was mainly expressed in the leaf rather than the other tissues (Figure 2a). *P. euphratica* plants were treated with exogenous ABA and drought stress to investigate the expression pattern of *PeCHYR1*. The results showed that the transcript abundance of *PeCHYR1* is transiently increased in the leaf under water stress, reaching a maximum of 3.5 times higher than control at 3 h, dropping to approximately 3 times at 6 and 9 h, and decreasing to 1.2 times at 12 h (Figure 2b). The abundance of *PeCHYR1* was gradually augmented by ABA treatment, significantly increasing at 0.5 h, maintaining a stable level until 6 h and reaching a maximum of 9 times higher than control at 12 h (Figure 2c).

Drought stress‐induced ABA generation leads to the accumulation of ROS. Young leaves of 1.5‐month‐old poplar were treated with 100 μm ABA for 0, 1, 2 and 3 h, and then, the leaves were immersed in diaminobenzidine (DAB) overnight. We observed that the red‐brown colour of the leaves darkened gradually as the time of ABA treatment progressed (Figure 2d).

### Subcellular localization of PeCHYR1

**Figure 3:**
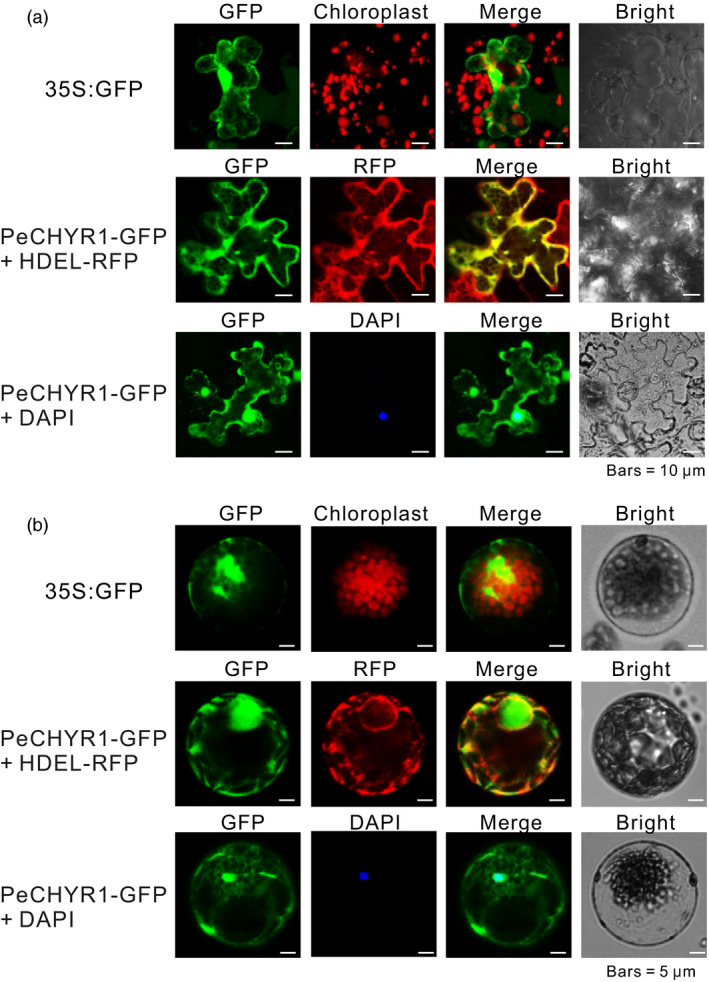
PeCHYR1 is targeted to the endoplasmic reticulum (ER) and nucleus. (a) Subcellular localization of 35S: GFP and 35S:PeCHYR1‐GFP in transiently expressed tobacco leaves. HDEL‐RFP was used as an ER localization marker fused with red fluorescent protein (RFP). The nuclear dye DAPI (blue) was applied to mark the nucleus. Bar = 10 μm. (b) Subcellular localization of 35S: GFP and 35S:PeCHYR1‐GFP in transiently expressed Arabidopsis leaf protoplasts. 35S: HDELRFP was cotransformed with 35S:PeCHYR1‐GFP to verify the ER localization of PeCHYR1. The nuclear dye DAPI (blue) was applied to mark the nucleus. Bars = 5 μm.

Previous studies indicated that Arabidopsis CHYR1, a ubiquitin E3 ligase, was generally localized in the nucleus, cytoplasm and endoplasmic reticulum (ER) (Ding *et al*., 2015). Thus, *P. euphratica* PeCHYR1, a functional homolog of CHYR1, might localize to the same cellular structures. To determine the subcellular localization of PeCHYR1, a 35S:PeCHYR1‐GFP (green fluorescent protein) fusion protein, together with a 35S: HDEL‐RFP (red fluorescent protein) fusion protein, was transiently transfected into tobacco leaves and Arabidopsis leaf protoplasts. Colocalization of PeCHYR1‐GFP and HDEL‐RFP was distinctly detected in the ER (Figure 3a,b). Simultaneously, colocalization with 4′, 6‐diamidino‐2‐phenylindole (DAPI) dye proved that the 35S:PeCHYR1‐GFP fusion protein was localized to the nucleus (Figure 3a,b). Overall, the results indicated that PeCHYR1 was localized to the nucleus and ER.

To study the potential biological functions of *PeCHYR1*, transgenic poplar 84K (*P. alba* ×* P. glandulosa*) plants overexpressing *PeCHYR1* were generated. Every transgenic line was verified by PCR, RT‐qPCR and histochemical staining with β‐glucuronidase (GUS) (Figure S2). In the literature, these three methods are usually used to verify transgenic plants (Jin *et al*., 2017).

### *PeCHYR1* promotes ABA‐induced stomatal closure via ROS production

**Figure 4:**
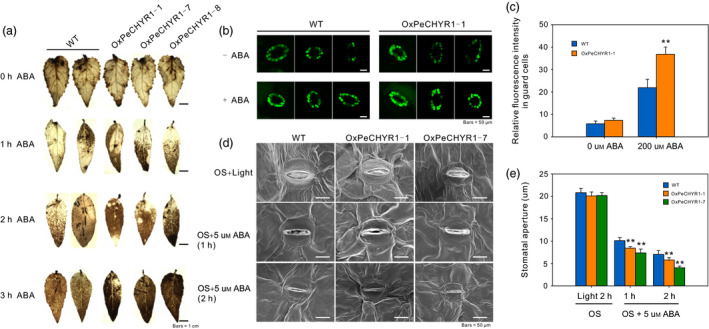
*PeCHYR1* promotes ABA‐induced stomatal closure via ROS production. (a) DAB staining shows different levels of ABA‐induced ROS production in the leaves of WT and 35S:*PeCHYR1*. Scale bars = 1 cm (b) Representative confocal images of 200 μm ABA‐induced H_2_O_2_ production (10 min) in the guard cells of WT poplar and transgenic lines coloured with H2DCFDA. (c) Quantification of H_2_O_2_ production in the guard cells of WT poplar and transgenic lines. (d) Detection of ABA‐induced stomatal closure in the leaves of the WT and transgenic lines; leaves were separated and immersed under light in stomata‐opening solution (OS) for 2 h and then treated with 5 μm ABA for 2 h (OS → ABA). (e) Stomatal closure was observed at 0, 1 and 2 h of ABA treatment with scanning electron microscopy of stomatal aperture. Scale bars = 50 μm. Error bars are means ± SE (*n *=* *50). Asterisks denote significant differences: **P <* *0.01.

Abscisic acid and ROS can induce stomatal closure (Bright *et al*., 2006), so we considered whether *PeCHYR1* could regulate ABA‐induced ROS accumulation. We treated 35S:*PeCHYR1* (*OXPeCHYR1‐1, OXPeCHYR1‐7, OXPeCHYR1‐8*) and WT plants with 100 μm ABA (0, 1, 2 and 3 h) and then performed DAB staining on their leaves. When the leaves of the different lines were treated with ABA, we found that the brown colour of 35S:*PeCHYR1* leaves was deeper than that of WT leaves (Figure 4a), even after multiple hours of treatment. These results indicated that 35S:*PeCHYR1* plants had higher ROS levels than WT plants in the instantaneous ABA treatment.

To confirm whether *PeCHYR1* participated in ABA‐induced H_2_O_2_ signalling, we measured the endogenous H_2_O_2_ levels in the stomata of WT and 35S:*PeCHYR1* poplars. We used 2,7‐dichlorodihydrofluorescein diacetate (H2DCF‐DA), a unique fluorescent probe, to detect the H_2_O_2_ levels within guard cells. In the absence of ABA, we found that 35S:*PeCHYR1* plants showed slightly elevated H_2_O_2_ content compared with WT plants (Figure 4b,c). In the presence of ABA, the levels of H_2_O_2_ were increased in both WT and 35S:*PeCHYR1* poplar, while 35S:*PeCHYR1* poplar exhibited significantly higher H_2_O_2_ levels than WT plants (Figure 4b,c).

To explore whether *PeCHYR1* promotes ABA‐induced stomatal closure, stomatal movement in response to ABA treatment was observed in WT and 35S:*PeCHYR1*. Then, leaf stomata were observed by scanning electron microscopy. Meanwhile, we observed a significant difference when the leaves were treated with 5 μm ABA for 1 and 2 h; the stomatal apertures closed more quickly in the leaves of the transgenic plants than in the leaves of the WT plants (Figure 4d,e). Conclusively, these data indicated that *PeCHYR1* actively regulated ABA‐induced ROS production.

### *PeCHYR1* overexpression enhances WUE by reducing stomatal conductance

**Figure 5:**
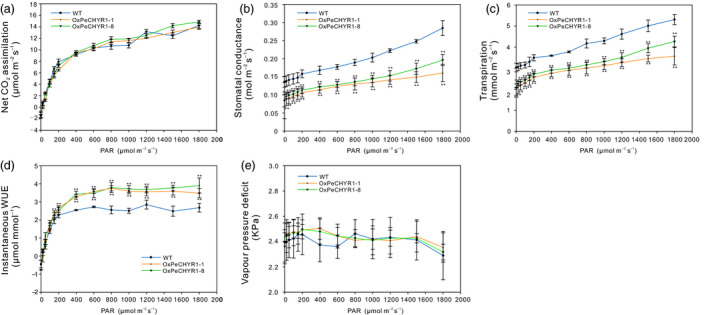
Light response curves were measured in WT, *OXPeCHYR1‐1* and *OXPeCHYR1‐8*. Light response curves were measured in the same greenhouse conditions. (a) A–light curve. (b) Gs–light curve. (c) Transpiration–light curve. (d) Instantaneous WUE–light curve. (e) VPD–light curve. Data are means ± SE (*n *=* *25). Asterisks denote significant differences: **P <* *0.01.

As H_2_O_2_ can also induce stomatal closure (Bright *et al*., 2006), we hypothesized that overexpression of *PeCHYR1* in transgenic poplar plants may decrease stomatal opening. To determine whether *PeCHYR1* could affect photosynthesis in plants, we measured photosynthesis–light curves in WT and 35S:*PeCHYR1* poplars. The data indicated that the patterns of photosynthesis were roughly similar in WT and 35S:*PeCHYR1* poplars (Figure 5a). The stomatal conductance (Gs) data indicated that WT and 35S:*PeCHYR1* poplars had altered Gs responses to light, while the Gs of 35S:*PeCHYR1* poplars was lower than that of WT (Figure 5b).

At the same time, leaf transpiration in 35S:*PeCHYR1* poplars was significantly lower than that in WT poplars (Figure 5c). In general, instantaneous WUE values in 35S:*PeCHYR1* plants were higher than those in WT plants (Figure 5d). Vapour pressure deficit (VPD) was measured, and no significant differences were found between WT and 35S:*PeCHYR1* poplars (Figure 5e). This lack of a significant difference in VPD indicates that the differences in transpiration rate were not caused by VPD.

### 35S:*PeCHYR1* poplars possess drought tolerance

**Figure 6:**
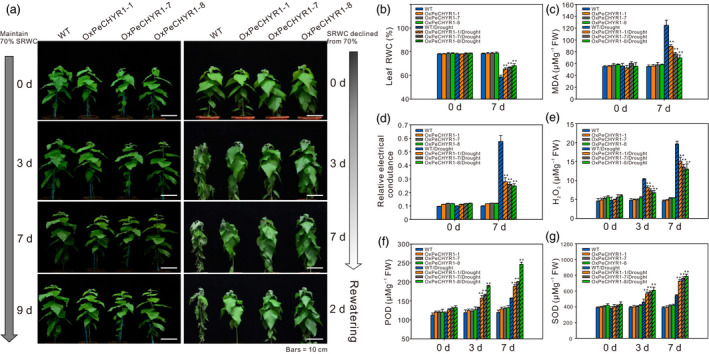
35S:*PeCHYR1* plants exhibited increased tolerance during short‐term drought treatment. (a) Morphological differences in short‐term drought assays. Bars = 10 cm. Quantitative measurement of leaf RWC (b), MDA content analysis (c), REC (relative electrical conductance) (d), H_2_O_2_ (e), activity of peroxidase (POD) (f), and activity of superoxide dismutase (SOD) (g) in the leaves of WT and 35S:*PeCHYR1* plants under normal and drought stress conditions. Values are means ± SE (*n *=* *40). All asterisks denote significant differences: **P <* *0.01.

Because ABA and drought stress can induce the expression of *PeCHYR1* (Figure 2b,c), we hypothesized that *PeCHYR1* played a vital role in drought response. Afterwards, 35S:*PeCHYR1* poplars and WT poplars were cultured under the same conditions and then subjected to a drought treatment in which the soil relative water content (RWC) was reduced from 70% and rewatered again for 2 days. On day 3, most leaves of the WT poplars were slightly wilted, while the transgenic poplars still appeared normal. On day 7, the leaves of the WT poplars were seriously wilted, while those of the transgenic poplars remained turgid. Furthermore, when the plants were watered again after drought stress, the WT poplars were unable to recover completely, whereas the transgenic poplars returned to normal and continued to grow (Figure 6a). Control plants were kept under the same conditions, except that the soil RWC was maintained at 70%, and no significant difference in physiological indicators and phenotype was found between 35S:*PeCHYR1* poplars and WT poplars in control conditions (Figure 6).

Leaf RWC reflects the degree of water deficit imposed. When exposed to short‐term drought stress, 35S:*PeCHYR1* poplar retained observably higher leaf RWC values than WT poplar (Figure 6b). As malondialdehyde (MDA) content is a valid indicator of cytomembrane oxidative damage, we measured the variations in MDA content caused by drought stress in the leaves of WT and transgenic poplars. The results indicated that the 35S:*PeCHYR1* poplars had lower levels of MDA than the WT poplars did when exposed to short‐term drought stress (Figure 6c). The REC (relative electrical conductance) of both 35S:*PeCHYR1* and WT poplars increased after drought treatment. After drought stress, the transgenic poplars showed less membrane damage (approximately 24.8%–27.9% ion leakage), whereas the leaves of WT poplars showed severe membrane damage (approximately 57.6% ion leakage) (Figure 6d).

To study the effects of drought stress in regard to H_2_O_2_, we measured H_2_O_2_ contents after 0, 3 and 7 days of drought stress. After withholding water for 0 days, the H_2_O_2_ contents in transgenic poplars were slightly higher than those in WT plants. The 35S:*PeCHYR1* transgenic and WT poplars both had increased H_2_O_2_ levels after 3 and 7 days of drought stress; however, the H_2_O_2_ content in WT poplars was significantly higher than that in transgenic poplars (Figure 6e). Peroxidase (POD) and superoxide dismutase (SOD) are crucial antioxidants that can scavenge ROS. Thus, we detected the activities of these two enzymes in 35S:*PeCHYR1* and WT poplars before and after drought treatment. Corresponded with the H_2_O_2_ levels, the results indicated that the activity levels of POD and SOD were dramatically elevated in 35S:*PeCHYR1* plants in comparison with WT plants under drought stress conditions (Figure 6f,g).

**Figure 7:**
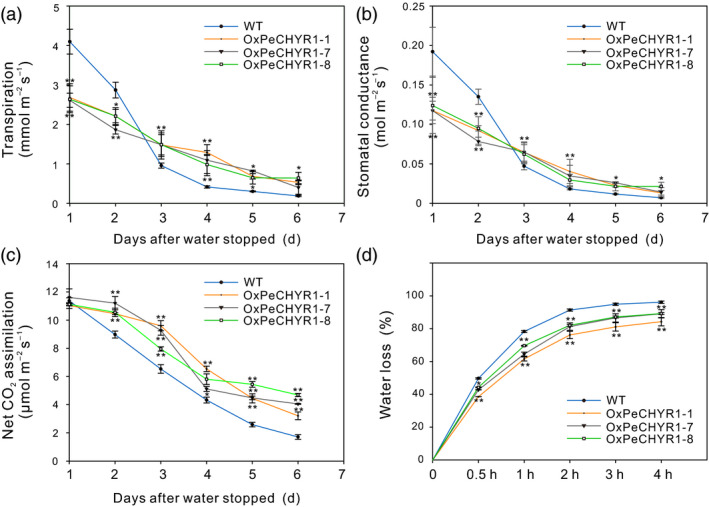
Variation in photosynthetic parameters of *35S:PeCHYR1* plants relative to those of WT plants during drought treatment and changes in the water loss rate of 35S:*PeCHYR1* plants relative to that of WT plants during natural dehydration. (a) Transpiration–drought time curve. (b) Gs–drought time curve. (c) A–drought time curve. (d) Water loss from detached leaves is indicated as a percentage of initial fresh weight. Values are means ± SE (*n *=* *25). All asterisks denote significant differences: *P <* *0.05; **P <* *0.01.

As shown in Figure 7a–c, the 35S:*PeCHYR1* transgenic and WT poplars during the 6 days of drought stress had remarkable variations in photosynthesis, Gs and transpiration. During these 6 days, photosynthesis in the transgenic and wild‐type poplars both decreased, while photosynthesis in WT poplars decreased more rapidly than that in the transgenic poplars under drought conditions, and photosynthesis in WT poplars was lower than that in the transgenic poplars after 2 days of drought treatment (Figure 7c). As with Gs, transpiration in the transgenic and WT poplars showed an overall decreasing trend, while transpiration in WT poplars decreased faster than that in transgenic poplars under drought conditions, and transpiration in WT poplars was lower than that in transgenic poplars after 3 days of drought stress (Figure 7a,b). Moreover, the data indicated that isolated leaves from WT poplars lose water faster than those from transgenic poplars under natural dehydration (Figure 7d). Conclusively, 35S:*PeCHYR1* transgenic poplars had better tolerance to drought stress.

### 35S:*PeCHYR1* poplars exhibited enhanced drought tolerance under long‐term water deficit stress

**Figure 8:**
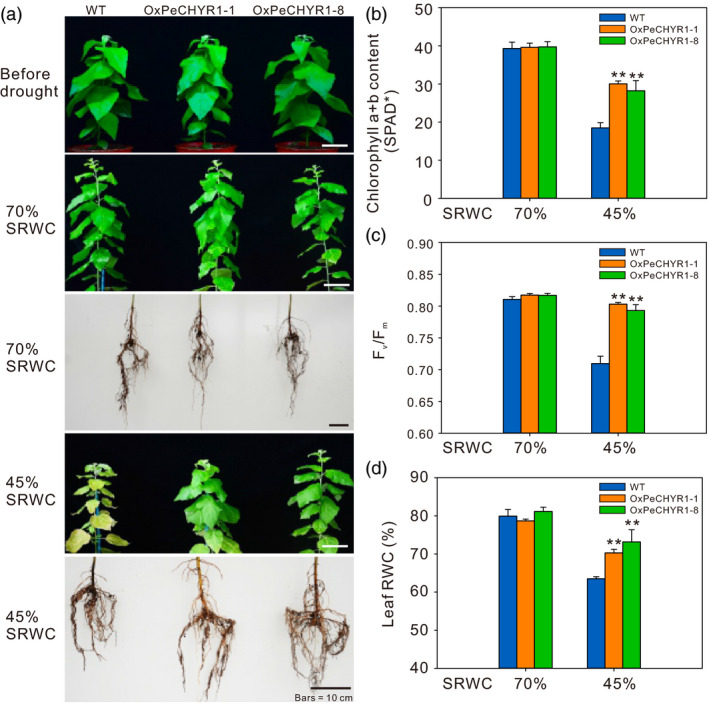
35S:*PeCHYR1* poplars exhibited high drought tolerance during long‐term drought. (a) Morphological differences between WT and transgenic plants during long‐term drought assays. Bars = 10 cm. The contents of chlorophyll a and b (b), Maximal PSII quantum yield (Fv/Fm) (c), and leaf relative water content (RWC) (d) under 70% soil RWC and 45% soil RWC. Data are means ± SE (*n *=* *40). Asterisks denote significant differences: **P <* *0.01.

To thoroughly study how *PeCHYR1* expression in plants influences the response to long‐term water deficit, 35S:*PeCHYR1* poplars and WT poplars were subjected to a 32‐day water‐deficit assay by holding the soil RWC at stable values (45% and 70%). After 32 days, in both shoot and root, the conditions of the WT and 35S:*PeCHYR1* poplar were approximately identical at 70% soil RWC. After 32 days of drought treatment, 35S:*PeCHYR1* poplar reached greater heights and appeared in better condition than WT at 45% soil RWC (Figure 8a). Furthermore, at 70% soil RWC, the chlorophyll level of 35S:*PeCHYR1* poplar was similar to that of WT plants. However, after the 32‐day water‐deficit assay at 45% soil RWC, 35S:*PeCHYR1* poplar had higher chlorophyll content than WT plants (Figure 8b). In addition, 35S:*PeCHYR1* poplar showed higher Maximal PSII quantum yield (Fv/Fm) and higher leaf RWC than WT poplar did (Figure 8c,d).

**Figure 9:**
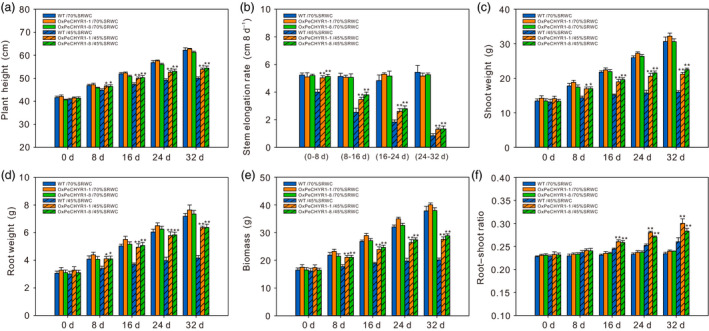
*PeCHYR1* positively regulates plant height and biomass increase under long‐term drought. (a) Plant height under 70% soil RWC and 45% soil RWC. (b) Stem height growth rate under 70% soil RWC and 45% soil RWC. (c) Shoot biomass, (d) root biomass, (e) whole plant biomass, and (f) root–shoot ratio in WT and 35S:*PeCHYR1* poplars under no water stress and mild water stress. Data are means ± SE (*n *=* *25). Asterisks denote significant differences: *P <* *0.05; **P <* *0.01.

To explore whether *PeCHYR1* would affect growth and development in the absence of water stress (70% soil RWC) and under mild water stress (45% soil RWC), plant height and stem height growth rate were monitored. The 35S:*PeCHYR1* poplar had similar plant height and stem height growth rate to those of wild‐type plants in the absence of water stress (Figure 9a,b). At 45% soil RWC, the 35S:*PeCHYR1* poplar had a significantly greater plant height and stem height growth rate than those of WT plants (Figure 9a,b). These results suggested that 35S:*PeCHYR1* poplar maintained higher growth under long‐term water‐deficit conditions.

To observe the difference in biomass accumulation between WT and 35S:*PeCHYR1* poplar under no water stress (70% soil RWC) and mild water stress (45% soil RWC), shoot weight, root weight, total biomass, and root–shoot ratio were monitored every 8 days. Under no water stress, transgenic and WT plants had similar biomass accumulations in all parts of the plant (Figure 9c–f). However, compared with that of WT plants, transgenic plants had more biomass accumulation in the shoot and root under mild water stress, and the shoot weight, root weight and total biomass of 35S:*PeCHYR1* poplar were obviously higher than those of WT plants after 8 days of drought treatment (Figure 9c–e). The root–shoot ratio reflects the relationship between the aboveground and underground parts of the plant. Both 35S:*PeCHYR1* and WT plants had greater root–shoot ratios, and 35S:*PeCHYR1* poplar had a larger root–shoot ratio than that of WT after 16 days of drought treatment (Figure 9f). After 32 days of drought treatment, the biomass of 35S:*PeCHYR1* poplar was 36.9 and 43.4% higher, respectively, than that of WT (Figure 9e), and the root–shoot ratio of 35S:*PeCHYR1* poplar was 15.3% and 8.9% higher, respectively, than that of WT (Figure 9f). In summary, under water‐deficit conditions, 35S:*PeCHYR1* poplar maintained higher growth than the WT.

### *PeCHYR1* mediated the expression of upstream and downstream genes involved in adversity

**Figure 10:**
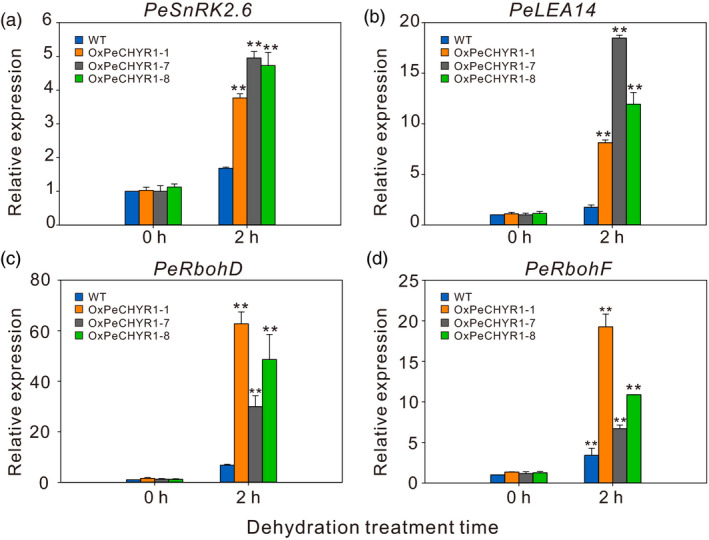
Stress‐responsive genes expressed in WT and 35S:*PeCHYR1* poplar plants under nonstress conditions and instantaneous drought stress conditions. Relative expression levels of *PeSnRK2.6* (a), *PeLEA14* (b), *PeRbohD* (c) and *PeRbohF* (d) in WT and 35S:*PeCHYR1* poplar plants under nonstress conditions and 2 h of drought stress. Total RNA was extracted from 2‐week‐old seedlings treated with drought stress for 2 h, and RT‐qPCR was performed. Data are means ± SE (*n *=* *20). Asterisks denote significant differences **P <* *0.01.

The genes *LEA14*,* RbohD*,* RbohF* and *Snrk2.6* are known to be drought responsive and have positive functions in drought tolerance (Ding *et al*., 2015). Thus, WT and 35S:*PeCHYR1* poplars were treated with 2 h of dehydration stress to investigate changes in *PeLEA14, PeRbohD, PeRbohF* and *PeSnRK2.6* expression. The results showed that the expression levels of *PeLEA14, PeRbohD, PeRbohF* and *PeSnRK2.6* in transgenic poplars were greater than those in WT poplars after 2 h of dehydration stress (Figure 10a–d). These results indicated that the improved drought tolerance of 35S:*PeCHYR1* transgenic poplars may depend on the regulation of downstream genes.

## Discussion

*PeCHYR1* was cloned from *P. euphratica*. A multiple alignment of amino acid sequences showed that *PeCHYR1, PtrCHYR1*, SpCHYR1 and *AtCHYR1* all contain a RING domain and a CHY zinc‐finger domain (Figure 1b). The conserved ‘CxHY’ motif lies in the CHY zinc‐finger domain, which plays a role in physiological interaction and ubiquitination (Lee and Kim, 2011; Leng *et al*., 2003) and consists of 12 histidines and cysteines that bind with three zinc ions, composing a unique zinc‐finger motif (Cayrol *et al*., 2007). Phylogenetic analysis indicated that the sequences of *PeCHYR1, PtrCHYR1* and *AtCHYR1* had higher homology (Figure 1a), potentially sharing a conserved and crucial function. Based on the evolutionary tree of related genes, *PeCHYR1, PtCHYR1* (Potri.009G005700) and *AtCHYR1* (AT5G22920) belonged to the first part (I) in the same branch (Figure 1a), which may decrease relative stomatal aperture and enhance drought tolerance under stress control conditions (Ding *et al*., 2015). However, *OsRZFP34, AtCHYR2* (AT5G25560) and *PtCHYR2* (Potri.006G245400) belonged to the third part (III) in the same branch (Figure 1a), which may control stomatal opening even with ABA treatment (Hsu *et al*., 2014). *PeCHYR1* also possesses a particular function; this protein is probably phosphorylated by *PeSnRK2.6* and augments drought and ABA‐induced responses in plants, which is similar to the function of *CHYR1* in Arabidopsis (Ding *et al*., 2015). Furthermore, transgenic poplar plants overexpressing *PeCHYR1* showed increased drought tolerance via ABA‐induced stomatal closure and ROS production.

Previous studies have indicated that plants regulate ABA‐induced stomatal closure by ROS accumulation (Bright *et al*., 2006; Hua *et al*., 2012; Li *et al*., 2017). H_2_O_2_ is a ROS that acts as a second messenger in ABA signalling in guard cells (Bright *et al*., 2006; Dietz *et al*., 2016; Kohler *et al*., 2003). The content of H_2_O_2_ in plants increases considerably under treatment with exogenous ABA (Figure 2d). This increased H_2_O_2_ level is an early signalling event in ABA‐induced signal transduction (Bright *et al*., 2006; Dietz *et al*., 2016; Kreslavski *et al*., 2012). In addition, the level of H_2_O_2_ increased more under treatment with exogenous ABA in 35S:*PeCHYR1* transgenic plants than in WT plants (Figure 4a–c). The altered relative expression levels of *PeRbohD* and *PeRbohF* (Figure 10c,d), which are involved in producing H_2_O_2_, were in agreement with the above results. Overexpression of *PeCHYR1* augmented ABA responsiveness, thereby activating stress‐inducible gene expression. These results indicated that *PeCHYR1* may promote ABA‐induced H_2_O_2_ production.

Abscisic acid is involved in abiotic stress response and regulates the expression of many stress‐responsive genes (Danquah *et al*., 2014; Kobayashi *et al*., 2005; Zhu, 2016). Whether these genes are induced in response to exogenous ABA treatment depends on their involvement in the ABA‐independent or ABA‐dependent stress response pathways (Yang *et al*., 2011). The protein DST generates an increase in hydrogen peroxide and promotes stomatal closure by an ABA‐independent pathway (Huang *et al*., 2009). However, 35S:*OsASR5* plants were more sensitive to exogenous ABA treatment than WT plants, suggesting that OsASR5 was involved in an ABA‐dependent pathway (Li *et al*., 2017). In our study, treatment with exogenous ABA did observably affect the expression of *PeCHYR1* (Figure 2c), and significant differences were present between the stomatal apertures of the WT and 35S:*PeCHYR1* lines under ABA treatment (Figure 4d,e), demonstrating that *PeCHYR1* increased sensitivity to exogenous ABA and reduced stomatal aperture. At the same time, *PeCHYR1* also participates in biological stress responses (Figure S3a,b).

Stomata control gas exchange, and they can regulate transpiration by limiting water loss and affect photosynthesis by facilitating CO_2_ uptake (Hetherington and Woodward, 2003; Vahisalu *et al*., 2008). Stomatal conductance is a direct result of stomatal density and aperture (Bussis *et al*., 2006; Chaerle *et al*., 2005; Qin *et al*., 2016). In our study, changes in transpiration were consistent with changes in stomatal conductance. As same as stomatal conductance, transpiration in 35S:*PeCHYR1* plants was significantly lower than that in WT plants (Figure 5b,c). However, the change of photosynthesis in WT plants was roughly similar to that in 35S:*PeCHYR1* plants. Accordingly, the instantaneous WUE in the transgenic plants was elevated (Figure 5d), consistent with the normal‐temperature water loss rate of 35S:*PeCHYR1* plants, which showed less water loss than WT plants did (Figure 7d). Previous studies have indicated that the decreased stomatal conductance in 35S:*PdEPF1* plants would not necessarily lead to decreased photosynthesis under well‐watered conditions (Wang *et al*., 2016). Rubisco and ribulose‐1,5‐bisphosphate are nonstomatal factors that play roles in modulating photosynthetic rate (Pearcy and Seemann, 1990; Qin *et al*., 2016), and thus, stomatal conductance does not solely control changes in foliar photosynthetic rate (Niinemets, 2002). These results indicate that a modest decrease in stomatal conductance in 35S:*PeCHYR1* transgenic plants can increase WUE but not affect photosynthesis under unstressed conditions.

When the plants were in a strictly water‐controlled environment, the reduced stomatal conductance of the 35S:*PeCHYR1* transgenic plants possibly reduced water evaporation and played an important part in maintaining a higher leaf RWC (Figure 7b). Under ABA and dehydration treatment, the expression level of *PeCHYR1* gradually rose (Figure 2b,c), consistent with the characteristics of drought tolerance in 35S:*PeCHYR1* transgenic plants. The evidence indicated that 35S:*PeCHYR1* plants had a stronger drought tolerance phenotype than WT plants did. Under drought conditions, 35S:*PeCHYR1* transgenic plants could assimilate more carbon dioxide (Figure 7c). Generally, the level of H_2_O_2_ in plants can significantly increase under stress conditions (Bhattacharjee, 2012; Bright *et al*., 2006; de la Garma *et al*., 2015). Abrupt increases in plant H_2_O_2_ content are regarded as a marker of various stresses that can induce the antioxidant defence system (Ding *et al*., 2010; Jiang and Zhang, 2002). Nevertheless, exceedingly high levels of H_2_O_2_ can give rise to oxidative injury (Kuge *et al*., 2010; Wang *et al*., 2008). In our study, both in the short‐term ABA treatment and early in the drought treatment, the endogenous hydrogen peroxide levels in 35S:*PeCHYR1* transgenic plants suddenly increased and became higher than those in the WT plants (Figure 4a–c), thereby activating the native antioxidant system to cope with the stress. However, when the plants were in extreme drought conditions, the accumulation of H_2_O_2_ in the WT was significantly higher than that in the overexpression lines (Figure 6e), the stomatal conductance in the WT lines almost reached zero (Figure 7b), and photosynthesis was markedly lower than that in the overexpression lines (Figure 7c). The major ROS‐scavenging mechanisms include APX (ascorbate peroxidase), POD, SOD and CAT (catalase) (Ahmad *et al*., 2008; Dietz *et al*., 2016; Willekens *et al*., 1997), and 35S:*PeCHYR1* plants showed higher activity levels of POD and SOD under drought stress conditions. POD and SOD, which are beneficial to the maintenance of ROS levels during long‐term drought, were activated in transgenic plants (Figure 6f,g). Our observations demonstrated that 35S:*PeCHYR1* transgenic plants underwent a sudden, dramatic increase in H_2_O_2_, which activated the antioxidant system to reduce oxidative damage after drought treatment.

In addition, the level of MDA indirectly indicates the extent of membrane lipid peroxidation (Shi *et al*., 2013, 2014). In our research, drought stress led to mass accumulation of ROS in WT plants, elevating the content of MDA compared with that in 35S:*PeCHYR1* transgenic plants, seriously damaging membrane permeability, causing intracellular ion leakage, and thereby exacerbating plant senescence and death (Figure 6c,d). Thus, WT plants continued to receive more damage than transgenic plants, and we conclude that overexpression of *PeCHYR1* in plants is beneficial for drought tolerance.

Maximal PSII quantum yield (Fv/Fm), which reflects the potential maximum light energy conversion efficiency of plants, is 0.8–0.85 under normal conditions in the vast majority of higher plants (Kitajima and Butler, 1975). Photosystem II efficiency declines in plants under environmental stress (Ke *et al*., 2016; Winter and Lesch, 1992). Although drought stress affects plant photosystem II electron transport and reduces chlorophyll content, 35S:*PeCHYR1* poplar exhibited higher chlorophyll content and photosystem II efficiency than WT poplar did (Figure 8b,c), leading to less injury under water‐deficit conditions (Figure 8a). Furthermore, we found that leaf RWC in 35S:*PeCHYR1* poplar was obviously higher than that in WT poplar (Figure 8d), which also ensured that plant photosystem II could transport electrons properly and that plants could assimilate carbon dioxide normally under severe drought stress. Our experiment clearly demonstrated that poplars with decreased stomatal conductance could increase in plant height and biomass under water‐deficit conditions (Figure 9). 35S:*PeCHYR1* poplar showed decreased transpiration, increased photosynthetic rate, enhanced WUE, elevated plant height and boosted biomass under water‐deficit conditions. Drought is the primary abiotic stress responsible for negatively influencing poplar growth (Dash *et al*., 2017; Ke *et al*., 2016; Yin *et al*., 2004). We suggest that 35S:*PeCHYR1* poplar, a novel and drought‐tolerant forestry species, can improve WUE and increase biomass under drought stress.

In summary, a ubiquitin E3 ligase from *P. euphratica*,* PeCHYR1*, increased sensitivity to exogenous ABA, enhanced ROS production and reduced stomatal aperture, thereby reinforcing plant drought tolerance. Moreover, the up‐regulation of *PeCHYR1* reduced transpiration by decreasing the stomatal conductance without influencing photosynthesis under normal conditions. 35S:*PeCHYR1* poplar achieved higher photosynthesis and biomass accumulation along with reduced levels of MDA and REC under drought stress. Therefore, the *PeCHYR*1 gene family will be conducive to further exploration of the mechanisms of drought tolerance, and we obtained a novel line of poplars with increased drought tolerance.

## Experimental procedures

### Plant materials

One‐year‐old seedlings of *P. euphratica* with heights of 15 cm were obtained from Yuli County, Korla, Xinjiang province, China (41°19′37.06″ N, 86°15′44.78″ E) in March, which lies in a warm temperate zone and has a continental desert climate. The average annual rainfall is 43 mm, and the average annual evaporation is 2700 mm. One‐year‐old seedlings of *P. euphratica* were transplanted in individual pots (10 L) containing sandy soil (approximately 70% sand) and placed in a seed plot [light cycle: 16.0 h of light (06:00 am–10:00 pm); temperature (20–24 °C)] at Haidian, Beijing, China (40°000N, 116°200E; 49 m above sea level). Potted *P. euphratica* were watered on the basis of evaporation demand and irrigated with 1 L of Hoagland nutrient solution every 2 weeks for 2 months before treatment (Li *et al*., 2011; Wang *et al*., 2016).

To analyse gene expression, similarly grown seedlings of *P. euphratica* (40–50 cm high, with 30–40 leaves) were subjected to ABA treatment and dehydration. The seedlings were treated with a solution containing 200.0 μm ABA (Sigma, A1049) and 1 g TWEEN® 40 (Sigma, P1504‐500 ML), which was sprayed on the leaves of *P. euphratica*. Meanwhile, the control was treated with 1 g TWEEN® 40 in ultrapure water. Dehydration treatment was applied to seedlings that had been removed from the soil and exposed to air with 70% RH at 23 °C under dim light for 12 h (Ma *et al*., 2010; Wang *et al*., 2016). To obtain complete plants, we gently pulled out each seedling and carefully washed the roots to remove the soil. For every test, leaves were separated from plants at the given time periods and promptly immersed in liquid nitrogen. We collected different organs and tissues of 2‐month‐old *P. euphratica*, including YL, young leaf; AL, adult leaf; OL, old leaf; Pe, petiole; X, xylem; and R, root at the same time and immediately immersed them in liquid nitrogen.

### cDNA cloning of *PeCHYR1* from *P. euphratica*

Total RNA was extracted from the collected materials using the cetyltrimethylammonium bromide (CTAB) method (Chang *et al*., 1993). In the last step, potentially contaminating DNA was removed by treatment with DNase I. We used a NanoDrop 2000 Spectrophotometer (Thermo, West Palm Beach, FL) to measure the quality and quantity of RNA. Two micrograms of total RNA was used for the reverse transcription reaction with a Tiangen FastQuant RT Kit (with gDNase) (Qiagen, Düsseldorf, Germany) according to the protocol. The cDNA of *PeCHYR1* was cloned by PCR, and the primers are displayed in Table S1.

### Quantitative real‐time polymerase chain reaction (RT‐qPCR) analysis

Twenty microlitres of cDNA was diluted 1 : 10 with nuclease‐free water. Each reaction contained 10 μL SuperReal PreMix Plus (Tiangen Bio Inc., Beijing, China), 2 μL ROX Reference Dye (Qiagen), 1 μL cDNA [single‐stranded circular DNA (sscDNA), corresponding to 10 ng of total RNA], 5.8 μL nuclease‐free water and 0.6 μm of each primer. The cycling parameters were 95 °C for 15 min and then 45 cycles of 20 s at 95 °C and 60 s at 60 °C (Wang *et al*., 2014). RT‐qPCR was performed with the ABI StepOnePlus Real‐Time PCR System (ABI, Foster City, CA) according to the manufacturer's specifications. Each experiment was performed in 20 replicates (five biological replicates × four technical replicates), and all primers used are displayed in Table S1. We used the software tool Primer Premier 6 to design primers. We calculated the relative expression level of *PeCHYR1* by the ratio = (Et)_**Δ** *CT*t_/(Er)_**Δ** *CT*r_ method (Wang *et al*., 2014, 2016).

### Phylogenetic and domain analysis of *PeCHYR1*

Homologous amino acid sequences were obtained from the Phytozome database (http://www.phytozome.net/), and a phylogenetic tree of *PeCHYR1* was estimated with MEGA 7. DNAMAN was used to examine the amino acid sequences and confirm the conserved structures. The sequence data from this study can be found in the Phytozome database (http://www.phytozome.net/). The accession numbers of the genes used are displayed in Table S2.

### Plasmid construction and genetic transformation of poplar

The *PeCHYR1* sequence was inserted into the SmaI and SacI sites in the pCAMBIA‐1301 vector. The construct was transformed into *Agrobacterium tumefaciens* (*EHA105*) and then transformed into poplar 84K using the leaf disc method (Hsu *et al*., 2011; Yao *et al*., 2016). The leaves of poplar 84K were incubated on substrate (pH 5.80) containing 0.020 mg/L thidiazuron (TDZ), 0.50 mg/L 6‐benzylaminopurine (6‐BA), 0.050 mg/L 1‐naphthaleneacetic acid (NAA), 250 mg/L cefotaxime, 4 mg/L hygromycin and 0.80% (w/v) agar for shoot induction and selection. The regenerated shoots were individually detached from the callus and inserted into a rooting culture [1/2 MS substrate including 0.050 mg/L NAA, 250 mg/L cefotaxime, 150 mg/L hygromycin phosphotransferase and 0.9% (w/v) agar]. One‐month‐old regenerated seedlings were transplanted to small pots (12 cm length × 12 cm width × 12 cm height) with the same soil potting soil, turfy soil, and vermiculite 1 : 1 : 1) and then placed in a greenhouse [light cycle: 16.0 h of light, 8.0 h of dark; temperature: 24 °C (light)/20 °C (dark); relative humidity: 40%–45%].

### Subcellular localization

To determine the subcellular localization of *PeCHYR1*, 35S:PeCHYR1‐GFP fusion protein and 35S: HDEL‐RFP fusion protein were transiently transfected together into tobacco leaves by the previously published protocol (Cui *et al*., 2012). Cotransformation of 35S: HDEL‐RFP in combination with 35S:PeCHYR1‐GFP was also performed in *Arabidopsis* leaf protoplasts by means of polyethylene glycol (PEG) treatment (Yoo *et al*., 2007). HDEL‐RFP was used as an ER localization marker (Cui *et al*., 2012; Tian *et al*., 2013). The nuclear dye DAPI (10 mg/mL; Sigma‐Aldrich) was applied to mark the nucleus. The transformed protoplasts and tobacco leaves were observed by laser confocal fluorescence microscopy (Leica TCS SP8). The LAS‐AF software was used to record the images. The following lines of the argon ion laser were used: 488 nm for GFP, 584 nm for RFP, 380 nm for DAPI and 488 nm for chlorophyll. Fluorescence was detected at 495–515 nm for GFP, 575–590 nm for RFP, 430–450 nm for DAPI and 650 nm for chlorophyll.

### Molecular verification of transgenic plants

Total DNA was extracted from every line using the CTAB method (Jiao *et al*., 2015; Verbylaite *et al*., 2010). We identified transformation lines by PCR using a forward primer (CAMV 35S promoter) and reverse primer (*PeCHYR1*). RT‐qPCR was used for the analyses of *PeCHYR1* expression levels in different transgenic lines. Total RNA was obtained through the CTAB method from the leaves of WT and transgenic plants. Two micrograms of total RNA was used for the reverse transcription reaction with the Tiangen FastQuant RT Kit (with gDNase) (Qiagen, Düsseldorf, Germany), according to the manufacturer's instructions. qRT‐PCR was performed with the ABI StepOnePlus Real‐Time PCR System (ABI, Foster City, CA) according to the manufacturer's specifications. The internal control was a combination of two reference genes, *PeActin* and *PeUBQ*, which were screened previously (Bustin *et al*., 2009; Czechowski *et al*., 2005; Rao *et al*., 2013; Wang *et al*., 2014). The primers used are shown in Table S1.

### Histochemical staining of GUS activity

Histochemical staining of GUS was handled as described previously (Lee *et al*., 2010). Briefly, isolated leaves were immersed at 37.0 °C in a mix including 2 mm 5‐bromo‐4‐chloro‐3‐indolyl‐β‐d‐glucuronide, 0.1 m sodium phosphate buffer (pH 7.0), 0.50 mm each of potassium ferri‐ and ferrocyanide, 10.0 mm EDTA (pH 7.0) and 0.10% Triton X‐100 for 12 h. Then, the isolated leaves were incubated in 70% ethanol for 12 h and photographed.

### DAB staining

DAB staining was applied to detect the production of H_2_O_2_, as described previously (Ding *et al*., 2015). The leaves of 2‐month‐old transgenic and WT plants were sprayed with 100 μm ABA (Sigma, A1049) and 1 g TWEEN® 40 (Sigma, P1504‐500 ML) for 0, 1, 2 and 3 h and infiltrated in prepared 0.1 mg/mL DAB solution (Beyotime, ST033) in the dark for 8 h at 28 °C. Meanwhile, the controls were treated with 1 g TWEEN® 40 in ultrapure water. To remove chlorophyll after DAB staining, the stained leaves were incubated in a solution of 70% ethanol, and then, they were photographed.

### H_2_O_2_ detection in guard cells

A unique fluorescent dye, H2DCF‐DA (Sigma, D6883 HZB1212), was used for H_2_O_2_ detection in the guard cells of the WT and transgenic plants (Huang *et al*., 2009). To observe transient ABA responses, the leaves of two‐month‐old seedlings (WT, *OXPeCHYR1*) were immerged in 50 mm H2DCF‐DA for 12 min at 25 °C in the dark. Then, the leaves were steeped in liquid 1/2 MS and treated with 200 μm ABA. Confocal measurements were taken every 30 s for 5 min of ABA stress in xyt mode. The amount of H2DCF‐DA fluorescence in guard cells was measured with Image‐Pro Plus6 (Shi *et al*., 2013).

### Stomatal movement analysis

Stomatal movement tests were performed as described previously (Cui *et al*., 2015; Hsu *et al*., 2014; Li *et al*., 2017), with slight amendments. Leaves from two‐month‐old seedlings (WT and *OXPeCHYR1*) were detached into wire netting and immediately fixed in liquid nitrogen. Then, the fixed samples were immediately put into an −80 °C Ultra‐low Freeze Dryer (Biosafer‐18A, Jiangsu, China (Mainland)) and fully dried for 24 h. The fully dried sections were coated with gold using an Ion Sputter Coater (Eiko Engineering, IB‐2, Japan). All observations were collected using a scanning electron microscope (Hitachi S‐4700, Japan). For the ABA‐induced stomatal closure experiments, the same parts of the leaves were immersed in stomata‐opening solution (OS) containing 0.01 m KCl, 0.1 m CaCl_2_ and 0.01 mm MES‐KOH for 0.5 h in the dark and 2.0 h in the light, and then, 5 μm ABA was added. Then, stomata were fixed after 0, 1 and 2 h of ABA treatment. The stomatal images were collected using a Hitachi S‐4700 scanning electron microscope. More than 100 guard cells from each sample were measured to derive the stomatal aperture.

### Drought tolerance experiment

Fifteen 35S:*PeCHYR1* and five WT poplar plants grown in a greenhouse [light cycle: 16.0 h of light (06:00 am–10:00 pm); temperature (20 °C–24 °C)] were subjected to a short‐term drought experiment. All of the plants (height 18–20 cm) were grown in suitably sized pots (12 cm × 12 cm × 12 cm), and each pot had a tray. All of the plants encountered drought treatment, in which the soil RWC was reduced from 70%. The control plants were kept in the same conditions, except that the soil RWC was maintained at 70%. A soil RWC of 70% induces no stress and is the most suitable environment for plant growth (Wang *et al*., 2016).

### Physiological and biochemical analysis

A Li‐Cor portable photosynthesis meter (LI‐COR 6400) was applied to detect net photosynthetic rate, transpiration and stomatal conductance in the ninth to eleventh leaves of 35S:*PeCHYR1* and WT poplar plants. We used the LI‐COR 6400 portable photosynthesis analysis system to measure light curves under normal conditions of poplars grown in a greenhouse for 1.5 months. Twenty plants were measured. The experiment covered net CO_2_ assimilation (A), Gs, transpiration, VPD and instantaneous WUE (A/transpiration). Photosynthetic light response curves were determined at photosynthetically active radiation (PAR) levels of 1500, 1200, 1000, 800, 600, 400, 200, 150, 100, 80, 50, 20 and 0 μm/m^2^/s with 450 μm/mol external CO_2_, which was controlled by the LI‐COR 6400 (Wang *et al*., 2016).

### Measurement of H_2_O_2_ content and antioxidant enzyme activities

Based on the continuous changes of photosynthesis under drought stress, H_2_O_2_ and antioxidant enzyme activities were measured at 0, 3 and 7 days. Samples of 0.5 g fresh leaves were detached from the drought treatment and control plants at 0, 3 and 7 days and ground into a fine powder in liquid nitrogen. The plant extracts were isolated in 50 mm sodium phosphate buffer (pH 7.8) mixed thoroughly with 1 mL of 0.1% (w/v) titanium sulphate (in 20%, v/v H_2_SO_4_) for 10 min (Shi *et al*., 2012, 2014). H_2_O_2_ content and the activities of antioxidant enzymes (SOD and POD) were measured using previously published protocols (Shi *et al*., 2014). The levels of H_2_O_2_, SOD and POD were expressed as μm (g * fresh weight (FW))^−1^. These experiments consisted of 20 replicates (4 biological replicates × 5 technical replicates) under identical conditions.

### MDA level and relative electrical conductance (REC) assay

Materials detached from drought‐stressed plants at 0 and 7 days were measured to determine MDA level and REC using previously published protocols (Shi *et al*., 2013, 2014). MDA (mol/g FW) = (6.45*(A532–A600) − 0.56 * A450) * 9 VtVs^−1^FW^−1^ [Vt: total volume of extract (mL); Vs: extract volume was determined with (mL)]. The electrical conductivity of the supernatant (L1) was detected using a DDS‐307 Conductivity Meter (Leici‐DDS‐307A, Shanghai, China). These experiments were independently replicated 6 times under identical conditions.

### Analysis of chlorophyll fluorescence and content

Photosynthetic activity in the 10th–13th leaves of transgenic and WT plants was monitored by Maximal PSII quantum yield (Fv/Fm) values, which reflect the potential maximum light energy conversion efficiency of plants, using a PAM chlorophyll fluorometer (PAM100) after 20 min of dark adaptation. Chlorophyll content was detected in the 10th–13th leaves of transgenic and WT plants using a portable chlorophyll meter (SPAD‐502Plus, Konica Minolta, Japan). Total chlorophyll contents were measured at 70% soil RWC and 45% soil RWC.

### Long‐term drought experiment

Drought stress was implemented in transgenic and WT poplar plants by maintaining the soil RWC at 45% for 32 days. Control plants were maintained at a soil RWC of 70%. A soil RWC of 70% causes no stress and is a suitable environment for plant growth (Wang *et al*., 2016). The transgenic and WT poplar plants (five poplars per line under various treatment) were grown in a greenhouse [light cycle: 16.0 h of light (06:00 am–10:00 pm); temperature (20 °C–24 °C)], all plants (height 40–43 cm) were kept in suitably sized pots (volume, 15 L), and every pot had a tray. The containers were weighed daily, and lost water was supplemented. We measured the height, shoot biomass and root biomass of each poplar every 7 days. After 32 days, chlorophyll content, leaf RWC and Maximal PSII quantum yield (Fv/Fm) were measured.

### Relative water content (RWC)

The 10th–13th leaves were detached and used for RWC measurements. In short, we determined the leaf FW (removed fresh leaves were weighed), leaf turgid weight (TW, leaves were measured after submerging in water for 8 h) and leaf dry weight (DW, leaves were measured after drying at 80 °C for 72 h). The RWC was calculated as (FW − DW)/(TW − DW) × 100%.

### Statistical analysis

The experimental data were subjected to analysis of variance by Statistical Product and Service Solutions 17.0 (SPSS). For statistical analyses, Student's *t*‐test was used to generate every *P* value (one‐way analysis of variance: *P <* *0.05; **P <* *0.01). Both one‐way and two‐way analyses of variance were used to determine significance. The data were normalized, and all samples were normally distributed with homogeneity of variance.

## Supporting information


**Figure S1** Analysis of Cis elements of the *PeCHYR1*gene.
**Figure S2** Analysis of the transgenic poplar plants overexpressing *PeCHYR1*.
**Figure S3** *35S: PeCHYR1* plants exhibited increased disease resistance under anthracnose fungus treatment.
**Table S1** Primer sequences used for cloning of *PeCHYR1* cDNA and RT‐PCR.
**Table S2** Accession numbers of gene.
