# Supplementary material for: PeCHYR1, a ubiquitin E3 ligase from Populus euphratica, enhances drought tolerance via ABA‐induced stomatal closure by ROS production in Populus
Source: Plant Biotechnol J. 2018 Mar 8;16(8):1514–28. doi: 10.1111/pbi.12893 (PMC6041450; doi:10.1111/pbi.12893)
Supplement: Supplementary file 1 — Figure S1 Analysis of Cis elements of the PeCHYR1gene. Figure S2 Analysis of the transgenic poplar plants overexpressing PeCHYR1. Figure S3 35S: PeCHYR1 plants exhibited increased disease resistance under anthracnose fungus treatment. Table S1 Primer sequences used for cloning of PeCHYR1 cDNA and RT‐PCR. Table S2 Accession numbers of gene. [file PBI-16-1514-s001.pdf]

Supplement materials

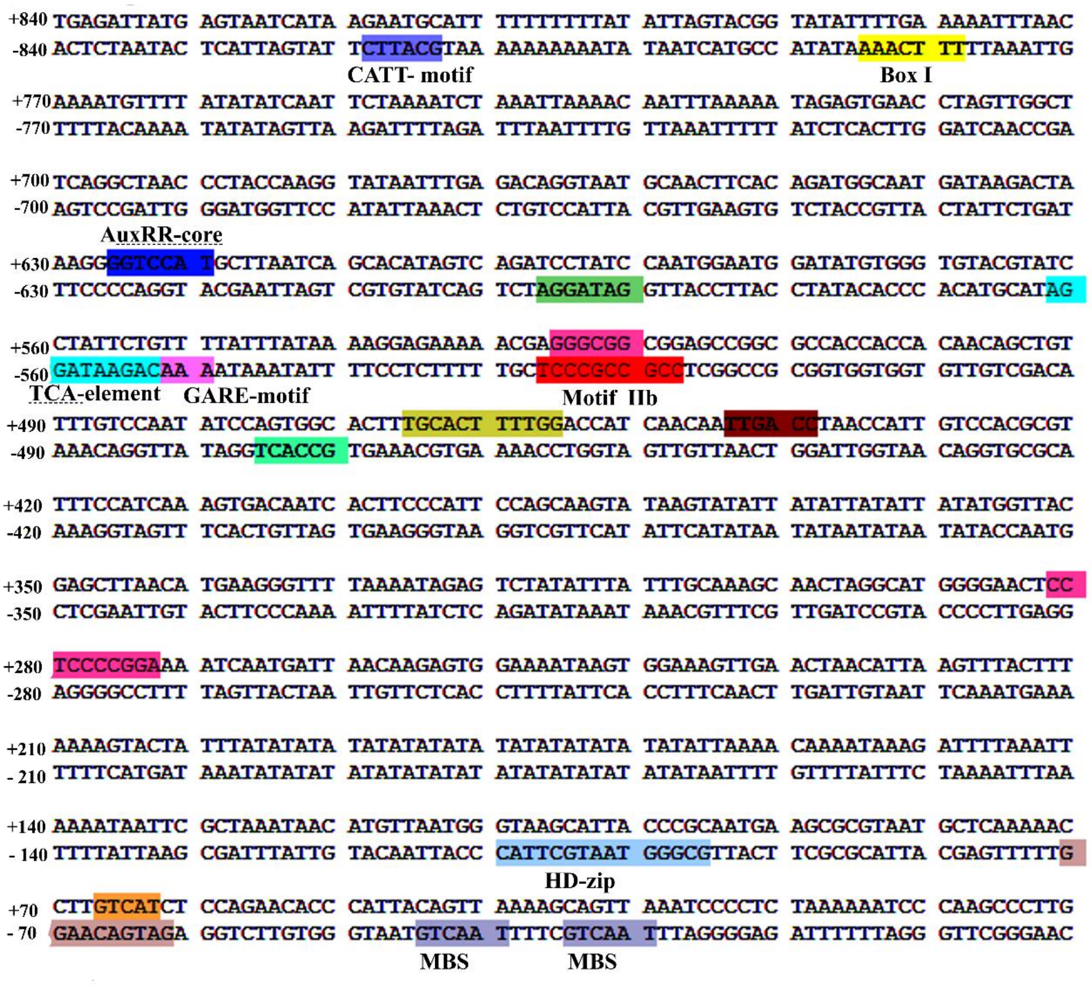

Figure S1 Analysis of Cis elements of the *PeCHYR1*gene.

The result suggested that the *PeCHYR1* promoter contains CAAT and TATA motifs. A prediction of the *PeCHYR1* promoter using the Plant CARE database (http://bioinformatics.psb.ugent.be)was performed to label a series of water-related stress-responsive, including one motif IIb (an abscisic acid-responsive element), two MBS (MYB binding site involved in drought responsiveness) elements, one AuxRR-core (an auxin-responsive element). An analysis of our qRT-PCR, and predictive results indicated that expression of *PeCHYR1* was induced in response to water deficit and ABA.

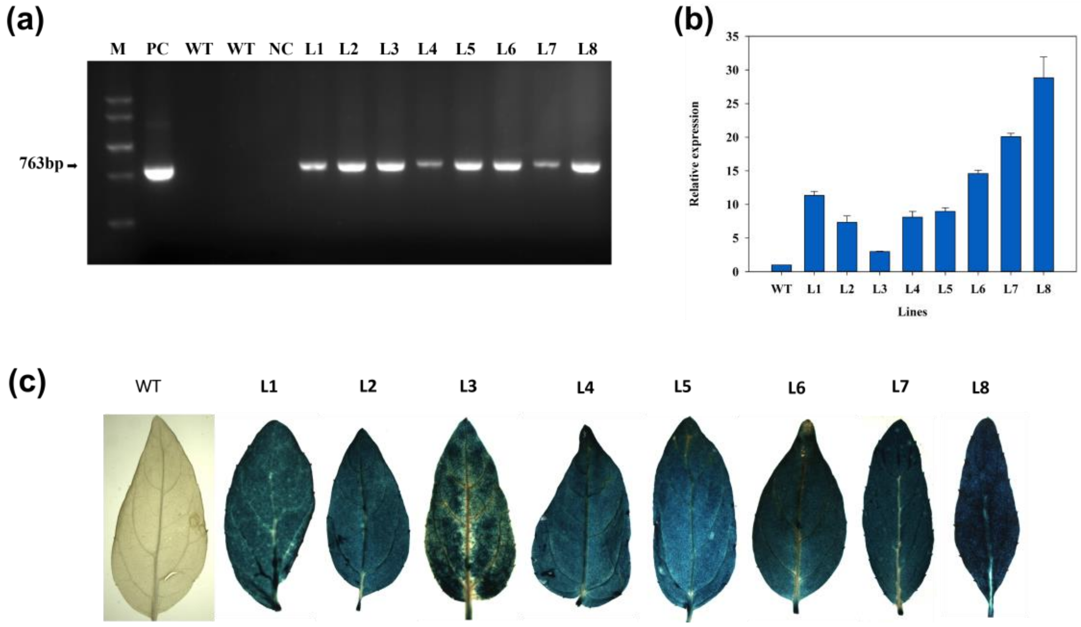

Figure S2 Analysis of the transgenic poplar plants overexpressing *PeCHYR1*. PCR analysis was handled in 8 putatively hygromycin-resistant poplars for the existence of *PeCHYR1* genes. (a) PCR confirmation of transgenic plants. 763bp PCR product was tested in 8 transgenic lines. M, λ-EcoT14 I digest DNA markers; PC, PCR product with pCAMBIA1301- *PeCHYR1* plasmid DNA as template; WT, wild-type , PCR product with wild-type plant genomic DNA as template; NC, negative control, PCR product with double-distilled water as template; L1–L8, PCR products with genomic DNA from regenerated hygromycin-resistant 84K leaves as template. (b) qRT-PCR analysis of *PeCHYR1* expression level in different transgenic lines. (c) Histochemical GUS staining of in different transgenic lines of 84K poplar. Error bars are means ±SE (n = 5).

Confirmation of 35S: *PeCHYR1*transgenic poplar

For exploring the role of *PeCHYR1* in drought tolerance, a construction containing *PeCHYR1* was introduced into the genome of the 84K poplar. At least 12 independently regenerated hygromycin-resistant lines were obtained, and eight transgenic lines (L1, L2, L3, L4, L5, L6, L7 and L8) were detected by PCR analyses. In eight transgenic lines we detected the predicted 763 bp

band (Figure S2a). The relative expression levels of *PeCHYR1* in these transgenic lines were different. Because transcript abundance of three independent transgenic lines (L1, L7, L8) were higher than other lines (Figure S2b), overexpression of *PeCHYR1* (OXPeCHYR1-1, OXPeCHYR1-7 and OXPeCHYR1-8) were selected for subsequent experiments. Furthermore, overexpression of *PeCHYR1* was verified by GUS staining assay in transgenic poplar. (Figure S2c).

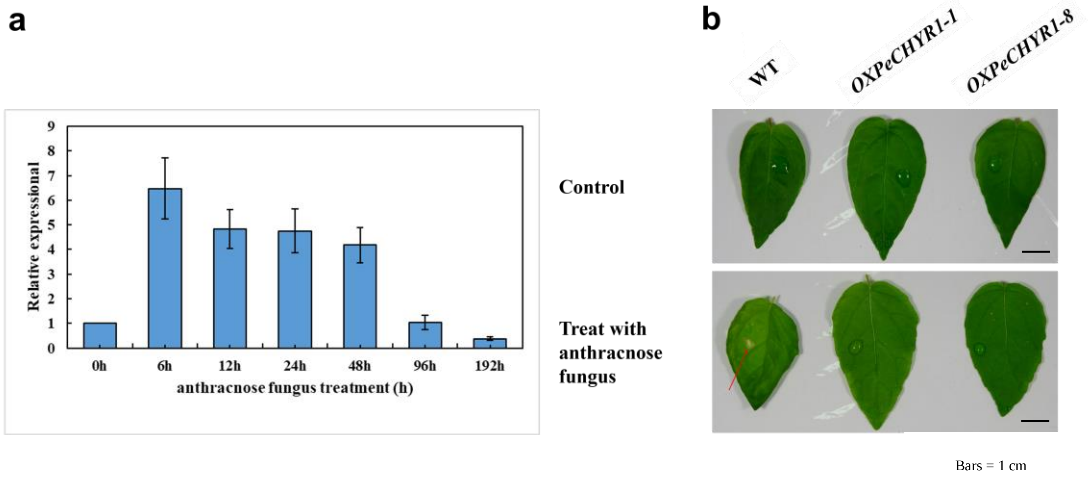

Figure S3. *35S: PeCHYR1* plants exhibited increased disease resistance under anthracnose fungus treatment. (a) The expression of PeCHYR1 was induced by anthracnose fungus (*Colletotrichum gloeosporioides*) treatment. The experiment was performed in three independent biological replicates and four technical repetition. (b) Pathogenicity test on detached poplar leaves.84K Poplar leaves incubated with the same amount of mycelial (*C. gloeosporioides*) were photographed 5 days after incubation. The control were treated with the same amount of ultrapure water.

Results

In order to know whether *PeCHYR1* have effects on disease resistance or susceptibility, quantitative real time PCR were handled to analysis of relative transcript abundance of *PeCHYR1* in *P. euphratica* plants which were treated with anthracnose fungus (*Colletotrichum gloeosporioides*) (Cannon et al., 2008). The results showed that the transcript abundance of *PeCHYR1* was induced by anthracnose fungus (*C. gloeosporioides*) treatment, reaching a maximum of 6.5 times at 6 h, holding on around 5 times at 12h, 24h, and 48h, turning into around 1 times at 96h, reducing to 0.4 times at 192h (Figure **S3a**).

To determine whether *PeCHYR1* was involved in *C. gloeosporioides* pathogenicity, the same

concentration of conidia was used to inoculate detached 84K poplar leaves of WT and transgenic line (*OXPeCHYR1-1 OXPeCHYR1-8*). WT of infection symptoms fully emerged 5 days after inoculation with conidia solution, but the leaves of transgenic line were not attacked after inoculation with conidia solution (Figure **S3b**).

Materials and methods

1. Fungal strains, culture conditions, and stress treatments

C. gloeosporioides strain BDL-3, isolated from *Populus×euramericana* Neva in Beijing, China, was used as the wild-type strain throughout this work. Both BDL-3 and its derivatives were cultured on PDA medium (200 g potato, 20 g glucose, 20 g agar, per 1 L of water) or in CM medium (1% glucose, 0.2% peptone, 0.1% yeast extract, 0.1% casamino acids, nitrate salts, trace elements, 0.01% vitamins, per 1 L of water, pH 6.5) for 3–15 days at 28° C to assess the growth and colony characteristics. Fungal mycelia were harvested from liquid CM and used for genomic DNA and RNA extractions. The strains were grown on PDA for conidiation assays. To analyzing expression of genes, similarly grown seedlings of *P. euphratica* (40–50 cm high, with 30–40 leaves) were subjected to anthracnose fungus (*Colletotrichum gloeosporioides*) treatment. The leaves of *P. euphratica* were handled with spore suspension (1×10^5) of the wild-type strain. For every test, leaves had been separated from plants at given time periods and promptly immersed in liquid nitrogen.

2. Quantitative real-time polymerase chain reaction (qRT-PCR) Analysis

Extractive of total RNA was obtained using the cetyltrimethyl ammonium bromide (CTAB) method from the collected materials (Azevedo et al., 2003). In the last step, potentially

contaminating DNA was obliterated by treatment with DNase I. We used a NanoDrop2000 Spectrophotometer (Thermo, West Palm Beach, FL) to measure the quality and quantity of RNA. 2μg of total RNA was used for the reverse transcription reaction by TIANGEN FastQuant RT Kit (with gDNase) (Qiagen, Düsseldorf, Germany) according to the protocol. 20μl of CDNA was diluted 1:10 with nuclease-free water. 20 μl Reactions contain 10 μl SuperReal PreMix Plus (TianGen Bio Inc., Beijing, China), 2 μl ROX Reference Dye (Qiagen), 1 μl cDNA [single-stranded circular DNA (sscDNA), corresponding to 10 ng of total RNA], 5.8μl nuclease-free water and 0.6 μM of each primer. Blank controls were run in triplicate for each master mix. The cycling parameters were: 95°C for 15 min, 45 cycles of 20 s at 95°C and 60 s at 60°C. qRT- PCR was operated with the ABI StepOnePlus Real-Time PCR System (ABI, Foster City, CA) according to the specification. Each experiment performed 20 replicates (five biological replicates X four technical replicates) and all primers used are displayed in Table S1. We used the software tool Primer Premier 6 to design primers. We calculated the relative expression level of *PeCHYR1* by the Ratio = $(Et)_{\Delta CTt}/(Er)_{\Delta CTr}$ method.

3. Pathogenicity test on detached poplar leaves.

Approximately 3-week-old detached 84K-poplar (*Populus alba*×*Populus glandulosa*) leaves of WT and transgenic line (*OXPeCHYR1-1 OXPeCHYR1-8*) were inoculated with spore suspension (1×10^5) of the wild-type strain. The control were treated with the same amount of ultrapure water. The inoculated tissues were incubated in a humidified chamber at 25 °C for 16 h of daylight. The disease lesions were recorded 5 day after inoculation. At least three virulence experiments were performed, with ten replicates each.

Discussion

Previous experiments revealed that overexpression of *PeCHYR1* enhanced drought tolerance by facilitating H_2O_2 production-mediated the pore closes in poplar. High concentration of ROS results in cellular damage or even hypersensitive cell death, whereas low concentration of ROS functions as developmental signal, controlling various aspect of plant biology (Ahmad et al., 2008; Dietz et al., 2016; Huang et al., 2016; Karuppanapandian et al., 2011; Pitzschke et al., 2006). The expression of *PeCHYR1* was induced by anthracnose fungus (*Colletotrichum gloeosporioides*) treatment (Figure A). The altered relative expression of *PeRbohD* and *PeRbohF* (Figure 10C, D), which are involved in producing H_2O_2, are in agreement with the above results. *35S: PeCHYR1* plants exhibited increased disease resistance during anthracnose fungus treatment. This requires the following job and experiments to understand the mechanism of plant disease resistance.

Table S1 Primer sequences used for cloning of PeCHYR1 cDNA and RT-PCR

Gene	Forward primers	Reverse primers
For cloning of *PeCHYR1* cDNA	5’ATGGGGAGTGAATCCGTG 3’	3’TCATTTAATCTCAATGCCTACTTC5’
For RT-PCR		
PeCHYR1	5’ATTCATGCCCAGTTTGCTCC3’	3’CTGGTACGTTTCAGGCATCG5’
PeSnrk2.3	5’TGGTGTGGCTAGGTTGATGA3’	3’CTGAAGCGACCAGCAATACA5’
PeSnrk2.6	5’ACGATGGAGACCGGTATGAG3’	3’ACGATGTTGGGATGCCTAAG5’
PeLEA14	5’GTCAAGACCCCGGAGGCTAC3’	3’GTCAAGACCCCGGAGGCTAC5’
PeRbohD	5’CAGTTCGGACACAGAAAGCA3’	3’TTCGGGATCCTCATTAGCAC5’
PeRbohF	5’AACTGGCTAGAGGGACTGAA3’	3’TGGATGGTGAGCAGGATGTA5’
PeActin2	5’ACTACCCTCCAATCCAGACACTG3’	3’TTGCTGACCGTATGAGCAAG5’
PeUBQ	5’ CGTGGAGGAATGCAGATTTT3’	3’ GATCTTGGCCTTCACGTTGT5’
For PCR identify transgenic lines	5’ACTACCCTCCAATCCAGACACT3’	3’CTGGTACGTTTCAGGCATCG5’

Table S2. Accession numbers of gene

Species	Name	**Accession numbers**
Populus euphratica	*PtCHYR1*	Potri.009G005700
Populus euphratica	*PtCHYR2*	Potri.006G245400
Populus euphratica	*PtCHYR3*	Potri.008G205400
Populus euphratica	*PtCHYR4*	Potri.014G134400
Amborella trichopoda	*AmTrCHYR1*	AmTr_v1.0_scaffold00102.5
Amborella trichopoda	*AmTrCHYR2*	AmTr_v1.0_scaffold00142.13
Amborella trichopoda	*AmTrCHYR3*	AmTr_v1.0_scaffold00057.172
Arabidopsis thaliana columbia	*AtCHYR1*	AT5G22920.1
Arabidopsis thaliana columbia	*AtCHYR2*	AT5G25560.2
Arabidopsis thaliana columbia	*AtCHYR3*	AT5G18650.1
Eucalyptus grandis	*EucgrCHYR1*	Eucgr.C01569.1
Eucalyptus grandis	*EucgrCHYR2*	Eucgr.C02874.1
Eucalyptus grandis	*EucgrCHYR3*	Eucgr.B01270.1
Eucalyptus grandis	*EucgrCHYR4*	Eucgr.H01203.1
Malus domestica	*MdCHYR3*	MDP0000125390
Malus domestica	*MdCHYR4*	MDP0000259643
Malus domestica	*MdCHYR2*	MDP0000242922
Malus domestica	*MdCHYR1*	MDP0000232929
Oryza sativa	*OsCHYR4*	Os12g35320
Oryza sativa	*OsCHYR2*	Os03g05270
Oryza sativa	*OsCHYR3*	Os03g22680
Oryza sativa	*OsCHYR1*	Os10g31850
Oryza sativa	*OsRZFP34*	Os01g52110
Prunus persica	*PpCHYR2*	Prupe.1G465000.1
Prunus persica	*PpCHYR3*	Prupe.1G141000.1
Prunus persica	*PpCHYR1*	Prupe.7G126100.1
Salix purpurea	*SpCHYR5*	SapurV1A.0375s0250.1
Salix purpurea	*SpCHYR4*	SapurV1A.1905s0020.1
Salix purpurea	*SpCHYR3*	SapurV1A.1059s0070.1
Salix purpurea	*SpCHYR2*	SapurV1A.1382s0050.1
Salix purpurea	*SpCHYR1*	SapurV1A.0288s0010.1
Salix purpurea	*SpCHYR6*	SapurV1A.0053s0100.1
Salix purpurea	*SpCHYR7*	SapurV1A.0022s0450.1
Theobroma cacao	*TcCHYR2*	Thecc1EG022296t1
Theobroma cacao	*TcCHYR3*	Thecc1EG038282t1
Theobroma cacao	*TcCHYR1*	Thecc1EG042163t1
Theobroma cacao	*TcCHYR4*	Thecc1EG021148t1
Zea mays	*ZmCHYR3*	Zm00008a013953_T01
Zea mays	*ZmCHYR1*	Zm00008a004267_T01

Zea mays	*ZmCHYR2*	Zm00008a000345_T01
Zea mays	*ZmCHYR4*	Zm00008a001478_T01
Zea mays	*ZmCHYR5*	Zm00008a036783_T01
Picea abies	*PaCHYR3*	MA_10436725p0010
Picea abies	*PaCHYR2*	MA_10429565p0010
Picea abies	*PaCHYR1*	MA_126866p0010
Pinus taeda	*PitaCHYR2*	PITA_000004179-RA
Pinus taeda	*PitaCHYR5*	PITA_000009259-RA
Pinus taeda	*PitaCHYR1*	PITA_000009261-RA
Pinus taeda	*PitaCHYR4*	PITA_000071111-RA
Pinus taeda	*PitaCHYR3*	PITA_000071113-RA
Pinus taeda	*PitaCHYR6*	PITA_000049143-RA
